# The evolving role of Fourier-transform mid-infrared spectroscopy in genetic improvement of dairy cattle

**DOI:** 10.1186/s40104-020-00445-2

**Published:** 2020-04-17

**Authors:** K. M. Tiplady, T. J. Lopdell, M. D. Littlejohn, D. J. Garrick

**Affiliations:** 1 Research and Development, Livestock Improvement Corporation, Private Bag 3016, Hamilton, 3240 New Zealand; 2 School of Agriculture, Massey University, Ruakura, Hamilton, 3240 New Zealand

**Keywords:** Bovine milk, Cattle breeding genetics, Fourier-transform infrared spectroscopy, Trait prediction

## Abstract

Over the last 100 years, significant advances have been made in the characterisation of milk composition for dairy cattle improvement programs. Technological progress has enabled a shift from labour intensive, on-farm collection and processing of samples that assess yield and fat levels in milk, to large-scale processing of samples through centralised laboratories, with the scope extended to include quantification of other traits. Fourier-transform mid-infrared (FT-MIR) spectroscopy has had a significant role in the transformation of milk composition phenotyping, with spectral-based predictions of major milk components already being widely used in milk payment and animal evaluation systems globally. Increasingly, there is interest in analysing the individual FT-MIR wavenumbers, and in utilising the FT-MIR data to predict other novel traits of importance to breeding programs. This includes traits related to the nutritional value of milk, the processability of milk into products such as cheese, and traits relevant to animal health and the environment. The ability to successfully incorporate these traits into breeding programs is dependent on the heritability of the FT-MIR predicted traits, and the genetic correlations between the FT-MIR predicted and actual trait values. Linking FT-MIR predicted traits to the underlying mutations responsible for their variation can be difficult because the phenotypic expression of these traits are a function of a diverse range of molecular and biological mechanisms that can obscure their genetic basis. The individual FT-MIR wavenumbers give insights into the chemical composition of milk and provide an additional layer of granularity that may assist with establishing causal links between the genome and observed phenotypes. Additionally, there are other molecular phenotypes such as those related to the metabolome, chromatin accessibility, and RNA editing that could improve our understanding of the underlying biological systems controlling traits of interest. Here we review topics of importance to phenotyping and genetic applications of FT-MIR spectra datasets, and discuss opportunities for consolidating FT-MIR datasets with other genomic and molecular data sources to improve future dairy cattle breeding programs.

## Introduction

Characterisation of milk composition in dairy cattle has a long history of scientific and commercial interest, with many countries establishing formal milk testing programs by the early 1900’s [1, 2]. Initial selection targets in these programs were yields of milk or fat, which were measured on a small scale from samples taken manually on farm. Over the course of the twentieth century, advances in refrigeration and transportation technologies, and the availability of automated on-farm milk meters, resulted in a shift to large-scale collection of samples, processed through centralised laboratories, with the scope extended to include quantification of traits such as protein yield and somatic cell counts. More recently, advances in analytical techniques have led to the widespread use of Fourier-transform mid-infrared (FT-MIR) spectroscopy for phenotyping major milk composition traits for dairy improvement programs.

Fourier-transform mid-infrared spectroscopy uses light from the mid-infrared region to scan milk samples and determine the presence of specific chemical bonds. Results are presented as an absorption profile, consisting of the absorbance values for individual infrared light wavenumbers across the mid-infrared region. Traits are predicted as a function of the individual FT-MIR wavenumber absorbances, enabling rapid, high-throughput phenotyping of milk traits such as fat and protein yields, at a fraction of the cost of estimating the components using other methods. Increasingly, there is interest in analysing the individual FT-MIR wavenumbers, and in utilising FT-MIR data to predict other novel traits of interest to the industry, because the spectra are already available as a by-product of routine milk testing. Many of these traits are relevant to consumer expectations and concerns about the nutritional quality of milk, and the impact of dairy production systems on animal health and the environment; and are also relevant to farmers as they seek to improve farming systems and select cows based on their productivity, reproductive performance and disease resistance.

**Fig. 1:**
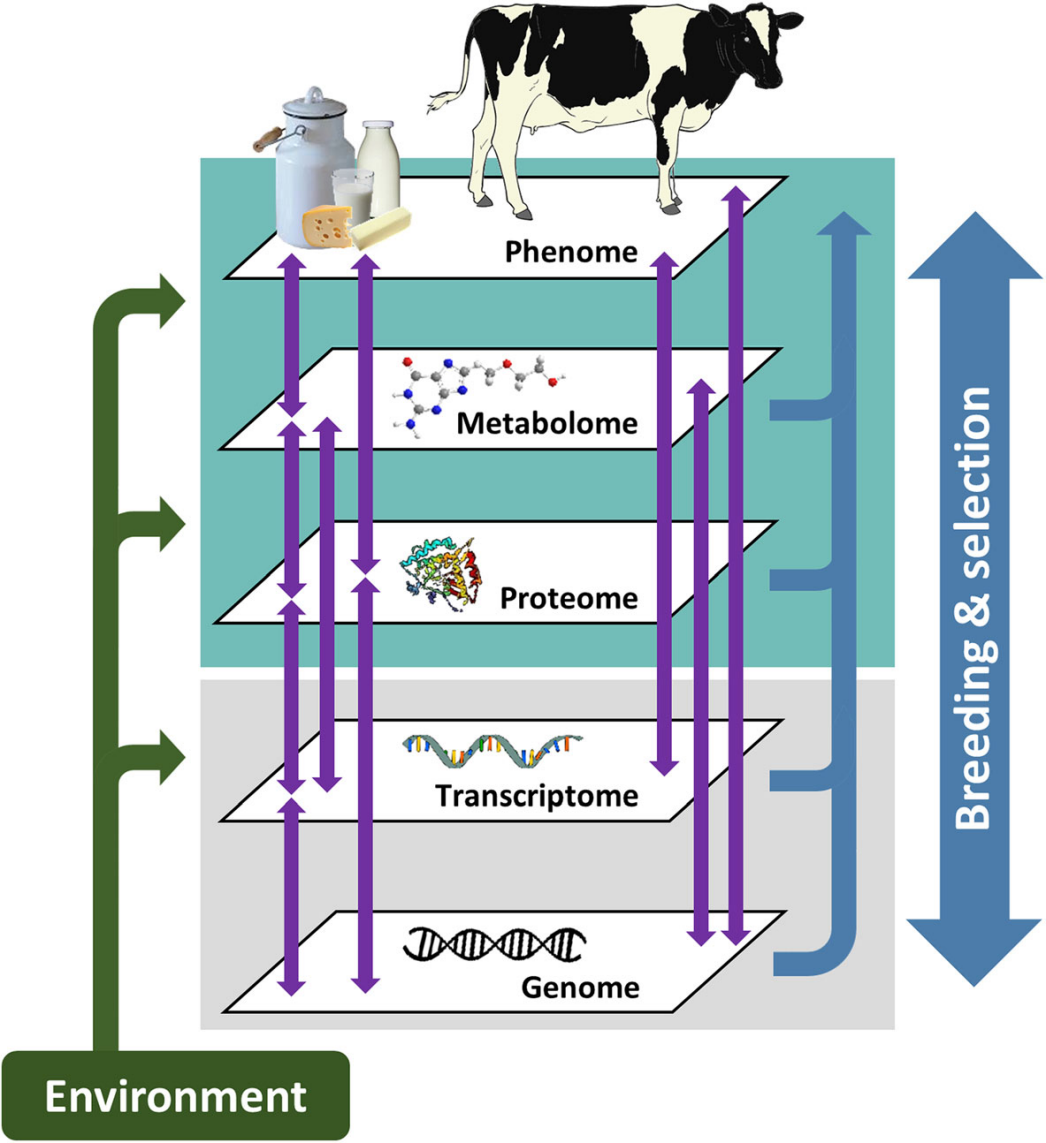
Characterisation of the relationships between molecular and biological mechanisms underlying phenotypic trait expression

Successful phenotyping using FT-MIR data is dependent on the magnitude of the phenotypic correlation between the predicted trait and the trait as measured by a benchmarked standard reference method. The successful incorporation of a FT-MIR predicted trait into a breeding program is further dependent on the heritability of the spectral-based predictions and on the genetic correlation between the spectral-based predictions and the trait as measured by the benchmarked standard [3, 4]. Improving our understanding of the genetics underlying the expression of FT-MIR predicted traits of interest is thus highly valuable. Conducting a genome wide association study (GWAS) is a widely used practice for identifying genomic regions that are influencing expression of complex traits, such as those predicted from FT-MIR data. However, linking complex traits, such as those predicted from FT-MIR spectra to specific genetic mechanisms is complex, as the phenotypic expression of traits are a function of a diverse range of molecular and biological mechanisms [5] that can obscure the underlying causal links between genotypes and phenotypes. These mechanisms may be characterised as a set of intermediate omics measures, including sugars, lipids and amino acids in the metabolome, proteins in the proteome, RNA molecules in the transcriptome and DNA in the genome, all of which interact with environmental factors to ultimately determine what is observed at the phenotypic level (Fig. 1).

Establishing causal links between the genome and observed phenotypes may be assisted by employing the individual FT-MIR wavenumbers, and other molecular phenotypes such as those related to the metabolome, chromatin accessibility, transcript levels, and RNA editing. Here we review the shifting role of FT-MIR datasets in dairy cattle improvement as we seek to predict new traits of importance to milk payment and animal evaluation systems. We discuss the broader topics of improving FT-MIR data quality and prediction model accuracy in phenotyping applications; and review existing studies of the genetics of FT-MIR predicted traits and individual FT-MIR wavenumbers. We also discuss opportunities for consolidating FT-MIR spectra datasets with other genomic and molecular data sources, to improve our knowledge of the genetic mechanisms of milk composition and enhance future dairy improvement programs.

### Phenotyping applications of FT-MIR spectra

Fourier-transform mid-infrared spectroscopy uses infrared light to scan a milk sample and determine the presence of specific chemical bonds. As the light passes through the sample, it interacts with the molecules present, causing vibrations and rotational changes in the molecular bonds, resulting in absorption of some of the light. The light absorption is typically represented as an absorption spectrum, consisting of the absorbance values for individual infrared light wavenumbers across the mid-infrared range. Traits of interest are subsequently predicted as a function of the individual FT-MIR wavenumber absorbances. Utilising FT-MIR data for the prediction of milk composition and other novel traits has been widely studied and recently reviewed [3, 4, 6, 7]. Other notable FT-MIR research includes studies of individual fatty acids and milk proteins [8, 9], and studies of milk properties related to manufacturing, especially coagulation and other cheese-making properties [10–12]. Further studies have focussed on traits not directly measurable in milk, including those related to pregnancy [13, 14], energy status [15, 16], nitrogen outputs [17] and methane emissions [18–20]. Such applications demonstrate that FT-MIR spectra can be used to predict a wide range of traits, including highly topical traits that are important to animal welfare and the environment. Whilst prediction accuracy is variable across these applications, a number of key principles and findings have been reported for improving spectra data quality and model prediction accuracy.

### FT-MIR data quality and prediction model accuracy

Trait prediction using FT-MIR spectra requires development of a calibration model, typically using a modest set of samples that have corresponding trait values, measured by a benchmarked technique. The most widely used method for developing calibration models from FT-MIR spectra has been partial least squares (PLS) regression. Fewer studies have employed Bayesian methods to develop calibration models [13, 21, 22], but no consensus has been attained as to which methodology is best at providing prediction accuracy [21–23]. This is likely due to the unique set of characteristics of each dataset, and indicates that it is advisable to assess a number of different modelling approaches for any given study. Once a calibration model is developed, the trait of interest can be estimated for any existing spectral absorbance data, or any future milk sample where the FT-MIR spectra data is available. The performance of a FT-MIR calibration model is assessed by how well the model predicts the benchmarked trait measurements in an independent dataset, or within the development dataset using a cross-validation framework. The utility and accuracy of trait predictions from FT-MIR spectra can often be improved by increasing the number of observations used to develop the calibration equation, and by ensuring that a similar extent of the variation in the prediction population is represented in the calibration dataset [24–27]. Prediction accuracy may also be improved by modifying the scale of the trait. For example, higher prediction accuracies have been reported when evaluating fatty acids as a percentage of total milk volume, compared to as a percentage of total fat content [24, 28, 29]. Similar considerations are important for studies of the concentrations of individual casein and whey proteins [28, 30–33]; and in studies related to cheese-making efficiency [28, 34–36]. Other considerations that influence prediction accuracy include: pre-processing treatments to address scaling and baseline effects in spectra data; appropriate management of outliers; low repeatability of sample measurement for specific regions of the infrared spectrum affected by the water content in milk; and managing systematic instrument variation due to factors such as temperature fluctuations and wavelength or detector intensity instability [37].

#### Pre-processing

Pre-processing treatments are commonly applied to FT-MIR spectra before generating a calibration model. The objective of pre-processing is to retain important discriminatory features of the spectra, but address baseline and scaling effects caused by light scattering, that can erode prediction accuracy. Baseline effects are additive and represent a baseline offset in the spectral response, whereas scaling effects are multiplicative and scale the spectral results by a given factor. One common group of methods for pre-processing are the multiplicative scatter methods [38, 39]. Multiplicative scatter correction is a normalization method that corrects spectra for scaling and baseline effects by comparing each spectrum to an expected spectral profile. Another family of techniques are the derivation methods, such as the Savitzky-Golay derivative [40]. Derivation methods are based on changes in the spectrum across specified window sizes, and are intended to smooth the spectrum whilst retaining key features of its shape.

Overall, there is no consensus about the best pre-processing treatment to apply to FT-MIR spectra. For example, some studies report that pre-processing spectra provides no significant gains to model prediction accuracy [35, 36], whilst others observe better predictions after pre-processing [27, 41], and several studies report mixed results [30, 33]. This is likely because of the unique characteristics of each dataset, indicating that in the development of a new calibration, it is advisable to compare a number of approaches to determine their effectiveness. Notably, even when different pre-processing strategies are examined in a study, authors often only report the best model, making it difficult to compare the effectiveness of other pre-processing strategies [3].

#### Outlier removal and removal of low signal-to-noise regions of the MIR spectrum

Outliers in FT-MIR datasets are often identified using a Mahalanobis distance (MD) metric, where the MD is a multivariate indicator of the distance between a spectral record and the average spectral response. Many studies are based on spectra from a single instrument, and are therefore not required to account for the different variance-covariance structures of measurements from different instruments. In a study of spectra from 66 instruments, Grelet et al. [42] showed considerable variability in the spectral responses of the instruments, while we have also observed that the distribution of MD values can be heterogeneous across instruments [43]. These results highlight the need to apply MD thresholds within instrument for the purpose of outlier removal.

Bands of the infrared spectrum with low repeatability of sample measurement due to the water content in milk are typically reported in the O–H bending (~ 1600 to 1700 cm^− 1^) and O–H stretching bands (>~ 3000 cm^− 1^). These regions have low signal-to-noise ratios, with varying boundaries reported across publications: 1600 to 1700 cm^− 1^ and 3040 to 3470 cm^− 1^ [30]; 1586 to 1698 cm^− 1^ and 3052 to 3669 cm^− 1^ [44]; 1600 to 1689 cm^− 1^ and 3008 to 5010 cm^− 1^ [42]. Although it is common practice to remove spectra from low signal-to-noise ratio regions, some studies indicate that there may be wavenumbers within these regions that carry valuable information. For example, Wang et al. [45, 46] identified wavenumbers in these regions that are affected by a polymorphism in the *DGAT1* gene, and Toledo-Alvarado et al. [13] identified a significant association between the 3683 cm^− 1^ wavenumber and pregnancy status. More generally, Bittante and Cecchinato [44] showed that the transmittance of individual spectra wavenumbers had moderate to high heritability across most of the mid-infrared region and highlighted that absorbance peaks for non-water milk components were present in low signal-to-noise ratio regions and should be considered for investigation. The findings of these studies indicate that a prudent approach to removal of wavenumbers in low signal-to-noise ratio regions should be taken, retaining spectra from all regions in applications where the wavenumbers are considered independently, but removing them in applications where wavenumbers are considered in a multivariate manner [43].

#### Managing systematic instrument variation

The instrument calibration approach outlined by Lynch et al. [47] has been widely used to standardize instrument predictions for major milk composition traits and reduce the impact of systematic variation between and within instruments across time. With this approach, a small set of reference samples are analysed through the instrument, where the reference samples have also been measured for traits of interest using benchmarked standards, such as the Rose-Gottlieb method for fat determination and the Kjeldahl method for protein determination. For these samples, unadjusted trait predictions are made from the spectra data, and instrument-specific correction coefficients are evaluated by comparing the unadjusted predictions to the measured trait values according to the benchmarked standard. A limitation of this approach is that it can only be used to adjust predictions for traits with pre-evaluated correction coefficients. More recent standardisation strategies have instead proposed calibrating the individual wavenumbers [42, 43, 48, 49], allowing the correction of any trait predicted as a function of the spectral wavenumbers. Studies have shown that standardising individual wavenumbers can effectively reduce prediction errors when transferring calibration models between instruments for fat composition traits [42, 48], as well as for calibration models for traits that are more difficult to predict reliably such as methane emissions and cheese yield [49].

Tiplady et al. [43] showed that the most consistent standardisation approach for reducing prediction errors relies on analysing identical reference samples across all instruments, as outlined by Grelet et al. [42]. Ideally, global reference sample sharing would be established, facilitating standardisation across instruments in different countries. That would enable the consolidation of spectral data collected on different instruments, and improve accuracy when applying calibration models developed on one instrument to spectral data collected on other instruments. Global reference sample sharing, however, is reliant on resolving issues related to sample preservation, and on adherence to the bio-security legislation of different countries. Instrument manufacturers such as Foss (Hillerød, Denmark) and Bentley (Chaska, MN) have started to offer alternative standardisation procedures. The Foss procedure uses a liquid equaliser with a known spectral response to adjust spectral results [50], whereas the Bentley procedure uses a polystyrene film to adjust for interferometer laser frequency shifts across time [51], and infrared flow cell information to adjust for shifts in absorbance measurement [52]. While these within-instrument standardisation procedures offer promise for automatic spectral standardisation, there have been no independent studies to validate their effectiveness for standardisation of milk samples collected across or within networks.

### The genetics of FT-MIR predicted traits

Predictions of major milk composition traits from FT-MIR spectra are already widely incorporated into dairy improvement programs. Other FT-MIR predicted traits that could be of interest to industry improvement programs include milk fatty acids and protein fractions, and traits that form proxy indicators for milk processability properties, and animal health and environmental outcomes. The accuracy of FT-MIR predictions is an important indicator of their utility, but for breeding purposes, the critical parameters are the extent of genetic variation in the benchmarked trait, the heritability of the FT-MIR predictions, and the genetic correlations between the FT-MIR predictions and the benchmarked trait.

#### Milk fatty acid and protein composition traits

Heritability estimates for FT-MIR predicted individual and grouped fatty acids, and their genetic correlations with gas chromatography (GC) based measurements are shown in Table 1. Where available, standard errors are shown in brackets. For individual milk fatty acids, heritability estimates ranged from 0.05 to 0.54 [9, 53, 54]. Heritability estimates for grouped fatty acids ranged from 0.11 to 0.51 [9, 55–57], with the lowest heritability estimates reported by Hein et al. [55]. In the studies by Fleming et al. [56] and Narayana et al. [57], heritability estimates were consistently higher for saturated fat and short- and medium-chain fatty acid groups, compared to unsaturated fat and long-chain fatty acid groups. Rutten et al. [54] was the only study to report genetic correlations between the FT-MIR predicted and GC-based fatty acids. These genetic correlations were high, ranging from 0.82 to 0.99.

**Table 1:** Heritability estimates for FT-MIR predicted fatty acids (*h*^2^), and their genetic correlations (*r*_*a*_) with GC-based^a^ fatty acids

Individual fatty acids^b^	Lopez-Villalobos et al. [9]	Soyeurt et al. [53]	Rutten et al. [54]	
	*h*^2^ (SE)	*h*^2^ (SE)	*h*^2^ (SE)	*r*_*a*_ (SE)
C4:0	0.38 (0.03)	–	0.42 (0.09)	0.94 (0.03)
C6:0	0.32 (0.03)	–	0.35 (0.09)	0.97 (0.02)
C8:0	0.29 (0.03)	–	0.38 (0.09)	0.99 (0.01)
C10:0	0.17 (0.02)	–	0.46 (0.10)	0.98 (0.01)
C10:1	0.30 (0.02)	–	–	–
C12:0	0.16 (0.02)	0.29 (0.02)	0.54 (0.11)	0.97 (0.02)
C12:1	0.41 (0.03)	–	–	–
C14:0	0.19 (0.02)	0.31 (0.03)	0.50 (0.10)	0.99 (0.01)
C14:1	0.27 (0.01)	–	–	–
C15:0	0.22 (0.02)	–	–	–
C16:0	0.29 (0.02)	0.38 (0.02)	0.30 (0.09)	0.86 (0.07)
C16:1	0.30 (0.02)	–	–	–
C17:0	0.41 (0.03)	–	–	–
C17:1	0.14 (0.02)	–	–	–
C18:0	0.26 (0.02)	0.30 (0.02)	0.52 (0.10)	0.82 (0.08)
C18:1	0.43 (0.03)	0.05 (0.01)	–	–
C18:1 *cis*-9	0.22 (0.02)	–	0.25 (0.08)	0.93 (0.05)
C18:1 *trans*-11	0.27 (0.03)	–	–	–
C18:2 *cis*-9, *cis*-12	0.45 (0.03)	0.20 (0.02)	–	–
C18:2 *cis*-9, *trans*-11	0.41 (0.03)	–	–	–
C20:0	0.38 (0.03)	–	–	–
C20:1 *cis*-11	0.37 (0.03)	–	–	–
C22:0	0.35 (0.03)	–	–	–
Grouped fatty acids^c^	Lopez-Villalobos et al. [9]	Hein et al. [55]	Fleming et al. [56]	Narayana et al. [57]
	*h*^2^ (SE)	*h*^2^	*h*^2^	*h*^2^
SCFA	0.39 (0.03)	0.16	0.42	0.24
MCFA	0.30 (0.03)	0.12	0.50	0.32
LCFA	0.50 (0.03)	0.11	0.26	0.23
SFA	0.46 (0.03)	0.15	0.51	0.33
UFA	0.48 (0.03)	–	0.26	0.21
PUFA	0.42 (0.03)	–	–	–

^a^*GC-based* Gas chromatography based.

^b^All fatty acids expressed as a % of the total fatty acids.

^c^*SCFA* Short-chain fatty acids, *MCFA* Medium-chain fatty acids, *LCFA* Long-chain fatty acids, *SFA* Saturated fatty acids, *UFA* Unsaturated fatty acids, *PUFA* Polyunsaturated fatty acids.

Fewer studies exist of the genetic parameter estimates of FT-MIR predicted individual milk proteins. Sanchez et al. [58] reported moderate to high heritability estimates (0.25 to 0.72) for a number of FT-MIR predicted milk protein contents/fractions (not shown), with especially high estimates for β-lactoglobulin (0.61 to 0.86). Moderate heritability estimates for FT-MIR predicted lactoferrin, ranging from 0.16 to 0.22 have also been reported [59–61]. These studies quantify the useful extent of genetic variation in FT-MIR predicted fatty acids and individual milk proteins, and suggest that these predicted traits could be incorporated into cattle improvement programs to change the fatty acid profile and the protein composition of bovine milk.

#### Milk processability traits

Heritability estimates and genetic correlations between measured and FT-MIR predicted milk processability traits are shown in Table 2. Where available, standard errors are shown in brackets. For coagulation traits, heritability estimates ranged from 0.16 to 0.43 [62–64]. Cecchinato et al. [63] was the only study reporting genetic correlations between FT-MIR predicted and measured coagulation traits (not shown). Those ranged from 0.91 to 0.96 for rennet coagulation time (RCT), and from 0.71 to 0.87 for curd firmness after 30 min (a_30_). Heritability estimates for FT-MIR predicted minerals ranged from 0.32 to 0.56, with phosphorus having the highest estimated heritability, and sodium having the lowest estimated heritability in both studies presented [64, 65]. Heritability estimates for nutrient recovery traits were typically higher than for cheese yield traits [66, 67]. Bittante et al. [66] was the only study reporting genetic correlations between FT-MIR predicted and measured cheese yield and nutrient recovery traits. These ranged from 0.76 to 0.98 for cheese yield traits, and from 0.79 to 0.98 for nutrient recovery traits. Overall, these studies show that many FT-MIR predicted processability traits are heritable, and that sufficient variation exists to use FT-MIR predicted traits to change milk processing and cheese-making characteristics in cattle improvement programs.

**Table 2:** Heritability estimates of FT-MIR predicted milk processability traits (*h*^2^), and their genetic correlations (*r*_*a*_) with measured traits

Trait^a^	Visentin et al. [62]	Cecchinato et al. [63]	Costa et al. [64]	Sanchez et al. [65]
	*h*^2^ (SE)	*h*^2^ range^b^ (SE)	*h*^2^ (SE)	*h*^2^ (SE)
Coagulation traits				
RCT, min	0.28 (0.01)	0.30–0.34 (0.08)	0.35 (0.05)	–
k_20_, min	0.43 (0.02)	–	0.43 (0.03)	–
a_30_, mm	0.36 (0.02)	0.22–0.27 (0.07)	0.39 (0.03)	–
a_60_, mm	0.27 (0.01)	–	–	–
HCT, min	0.16 (0.01)	–	–	–
CMS, nm	0.31 (0.02)	–	–	–
Acidity				
pH, units	0.27 (0.01)	–	–	0.37 (0.01)
Minerals, mg/kg milk				
Calcium	–	–	0.45 (0.02)	0.50 (0.01)
Phosphorus	–	–	0.53 (0.03)	0.56 (0.01)
Magnesium	–	–	0.47 (0.03)	0.52 (0.01)
Potassium	–	–	0.45 (0.03)	0.53 (0.01)
Sodium	–	–	0.38 (0.03)	0.32 (0.01)
	Sanchez et al. [65]	Bittante et al. [66]		Cecchinato et al. [67]
	*h*^2^ (SE)	*h*^2^ (SE)	*r*_*a*_	*h*^2^ range^c^
Cheese yield, %				
CY_CURD_	0.38 (0.01)	0.21 (0.09)	0.97	0.18–0.33
CY_SOLIDS_	0.39 (0.01)	0.22 (0.08)	0.98	0.18–0.28
CY_WATER_	–	0.18 (0.05)	0.76	0.14–0.29
Nutrient recovery, %				
REC_PROTEIN_	–	0.44 (0.09)	0.88	0.32–0.41
REC_FAT_	–	0.28 (0.07)	0.79	0.15–0.33
REC_ENERGY_	–	0.21 (0.07)	0.96	0.19–0.30
REC_SOLIDS_	–	0.24 (0.08)	0.98	0.17–0.29

^a^*RCT* Rennet coagulation time; k_20_ = curd-firming time; a_30_ = curd firmness after 30 min; a_60_ = curd firmness after 60 min; HCT = heat coagulation time; CMS = casein micelle size; CY: weight of fresh curd, curd solids, and curd as a percentage of weight of milk processed; REC: protein, fat, energy and solids of the curd as a percentage of the protein, fat, energy and solids of the milk processed.

^b^Range of estimates from 4 subsets of data used to validate calibration equations.

^c^Range of estimates from 3 different breeds.

#### Animal health traits

Health and fertility traits are valuable targets for breeding programs and selection for these traits would be considerably enhanced if they could be reliably predicted from FT-MIR. A recent review by Bastin et al. [68] across a wide range of FT-MIR predicted traits related to fertility, mastitis, ketosis and other disease traits highlighted that more research is required to understand the relationships between health and fertility indicators and FT-MIR predicted traits, and to estimate the genetic parameters of these traits. Since then, Belay et al. [69] have reported moderate heritability estimates for FT-MIR predicted blood β-hydroxybutyrate (BHB), ranging from 0.25 to 0.37 across different stages of lactation, and moderate genetic correlations between clinical ketosis and the FT-MIR predicted BHB (0.47). More research is required in this area to realise the value that FT-MIR spectra might add to animal health breeding goals.

#### Environment traits

Despite increasing interest in FT-MIR predictions of environmental traits related to methane (CH_4_) and nitrogen outputs from dairy systems, there have been few reports of the genetic parameter estimates of these FT-MIR predicted traits, or of the genetic correlations between measured and FT-MIR predicted trait values. Kandel et al. [70] report moderate heritability estimates, ranging from 0.22 to 0.25 for predicted daily CH_4_ emission and 0.17 to 0.18 for log-transformed predicted CH_4_ intensity. There is, therefore, some potential for the future incorporation of FT-MIR predicted methane traits into breeding programs. However, there are still issues to be resolved to address uncertainties and discrepancies in methane datasets and measurement methods, and to improve the accuracy and robustness of prediction equations to make them applicable across a broader range of production systems and environments [19, 20, 71, 72].

Milk urea nitrogen (MUN) concentrations are routinely predicted using FT-MIR spectroscopy [4], however, there are few studies of the genetic parameters of FT-MIR predicted MUN and its relationship with other production traits. Amongst those studies, moderate to high heritability estimates, ranging from 0.38 to 0.59 were reported by Wood et al. [73] and Miglior et al. [74], with lower estimates of 0.22 and 0.14 reported in studies by Mitchell et al. [75] and Stoop et al. [76], respectively. Mitchell et al. [75] was the only study reporting genetic correlations between wet-chemistry direct measurements of MUN and FT-MIR predicted MUN, which were 0.38 and 0.23 in lactations 1 and 2, respectively. These genetic correlations are significantly lower than those reported for fatty acids (0.82 to 0.99) [54] and milk processability traits (0.76 to 0.98) [66], and indicate that wet-chemistry measurements of MUN and FT-MIR predicted MUN are genetically different traits. Large differences in heritability estimates across studies of FT-MIR predicted MUN indicate that there may be underlying instability in prediction equations. This highlights the importance of developing prediction models that are robust across different breeds and production systems. Research is ongoing to determine the role that FT-MIR predicted MUN could have in reducing nitrogen outputs from dairy systems.

### The genetics of individual FT-MIR wavenumbers

In contrast to the prevalence of studies reporting genetic parameter estimates of FT-MIR predicted traits, there are relatively few studies reporting genetic parameter estimates for the individual spectral wavenumbers. Nevertheless, the transmittance of FT-MIR spectra wavenumbers is moderately to highly heritable across a large proportion of the mid-infrared region [44, 45, 77–79]. Although heritability estimates were consistently low in water absorption regions across all studies, estimates > 0.2 were reported across most of the mid-infrared region in studies by Soyeurt et al. [77] and Wang et al. [45]. This indicates that genetic gain may be obtained by directly selecting on a linear function of estimated breeding values (EBV) for individual FT-MIR wavenumbers; rather than indirect selection as currently practised on EBV of composite indicator traits, like fat yield, which are linear functions individual FT-MIR wavenumber absorbances. Recent studies have confirmed this, showing that the accuracies of breeding value predictions estimated directly from FT-MIR spectra can be higher than for breeding value predictions estimated indirectly from the FT-MIR predicted composite traits [80–82]. Estimating breeding values directly from FT-MIR spectra requires that spectral data is routinely stored, rather than just the spectral based predictions of milk components, and that has not historically been the case in most dairy nations.

### GWAS of individual FT-MIR wavenumbers

Many GWAS have been published in the last decade for FT-MIR predicted major milk production traits [83–87], and for fatty acid and protein fractions [88–92]. However, only two studies report GWAS results for individual FT-MIR wavenumbers. In a study of 1748 Dutch Holsteins across 50,688 SNP, Wang & Bovenhuis [46] conducted a GWAS on a subset of 50 wavenumbers, selected using a clustering approach to capture more than 95% of the phenotypic variation. In that study, significant associations between individual wavenumbers and over 20 genomic regions were identified. While most of these genomic regions had already been reported for having significant associations with other milk production traits, three new regions were identified. In a larger study of 5202 Holstein, Jersey and crossbred cows across 626,777 SNP, Benedet et al. [93] used a PLS approach to associate genotypes to spectral data, and showed that FT-MIR spectra could be used to increase the power of a GWAS, and assist with distinguishing milk composition QTL. The studies by Wang & Bovenhuis [46] and Benedet et al. [93] both demonstrate that there are genetic signals in the individual FT-MIR wavenumbers that we do not observe in the currently-used portfolio of composite FT-MIR predicted traits. This confirms that the individual FT-MIR wavenumbers can provide an additional layer of granularity to assist with establishing causal links between the genome and observed phenotypes. Notably, both studies use relatively low numbers of animals compared to recent GWAS published for other traits, and applying these methodologies to larger datasets, with higher genotype densities, promises to increase the power of these approaches. This should enable the discovery of QTL with smaller effect sizes in addition to novel QTL characterised by lower minor allele frequencies than those QTL discovered with datasets numbering only thousands of animals.

#### Computational challenges

Over the last two decades, the scope of genomic resources available for GWAS has increased, both in terms of the number of genotyped individuals, and in terms of variant density. Developing strategies for managing GWAS on large numbers of densely genotyped individuals is an active area of research, as we look to generate new, more efficient algorithms that will enable the processing of these datasets within acceptable timeframes and computational limits of RAM and CPU. The importance of efficient algorithms is further highlighted when we conduct GWAS across large numbers of FT-MIR predicted traits and the individual FT-MIR wavenumbers. Existing mixed-linear model-based methods for conducting GWAS, such as GCTA-MLMA [94] primarily run in *O* (*mn*^2^) or *O *(*m*^2^*n*) time per trait, where *m* is the number of variants and *n* is the number of animals. These models become prohibitively slow as the numbers of genotyped individuals and variants increase [95]. The ever-increasing cohort sizes of densely-genotyped individuals frequently requires subsampling to use these methods within acceptable computation constraints. This has spurred the development of faster, more memory-efficient algorithms and software. One software package, Bolt-LMM [95, 96] runs in approximately *O* (*mn*^1.5^) time; however, it makes assumptions that are valid only for larger sample sizes. Recent versions are capable of running the entire UK biobank data set (*n* = 459 k) in a few days on a single computational node [96]. Another algorithm, fastGWA [97], available as a recent enhancement of the GCTA software package, provides further reductions in algorithmic complexity, running in approximately *O* (*mn*) time. These improvements mean that it is capable of running *n* = 400 k UK biobank samples in around 20 min, compared to 22 h for BOLT-LMM on the same hardware. Developments such as this make GWAS across sizeable populations with large numbers of FT-MIR phenotypes feasible.

### Consolidating FT-MIR spectra with other omics data sources for QTL mapping

After conducting a GWAS, it is useful to identify the candidate genes and mutations underlying genomic loci with signal for a trait of interest. This can aid marker-assisted selection and improve our understanding of the biological pathways regulating the trait. Moreover, it has been shown that genomic prediction can be improved by including variants close to the causative mutations [98]. Software such as Ensembl’s Variant Effect Predictor [99] is commonly used to identify candidate causal variants that have protein-coding or loss-of-function effects, with the expectation that these variants are more likely to impact the trait than other variants. However, recent studies in both humans [100, 101] and dairy cattle [86, 102] have highlighted the prevalence of QTL underpinned by expression-based mechanisms, and demonstrate that the majority of variance for at least some traits can be explained by non-coding variants located in regulatory elements. These variants are typically identified by considering the expression levels of genes as phenotypes, and using these data for genetic mapping studies in an approach known as expression QTL (eQTL) analysis.

#### Expression-based phenotypes

Assuming a causality chain hypothesis, as illustrated in Fig. 1, observation of an eQTL, co-located with a QTL for an FT-MIR predicted trait can inform on the mechanism of the trait of interest. This methodology can also be used to identify mechanisms underlying QTL observed for individual FT-MIR wavenumbers. A strong correlation between the variant effects for the two QTL (expression and FT-MIR related) suggest a shared underlying genetic architecture regulating both, while a weak correlation suggests that the two QTL, though co-located, do not co-segregate, and therefore represent distinct genetic signals with different causal variants.

Similar to eQTL analysis, a range of additional omics data sources can be used for QTL mapping, and the resulting QTL could be applied to identify causative genes for FT-MIR predicted traits and individual FT-MIR wavenumbers. The factors yielding these omics data sources can occur before or after mRNA transcription. Factors acting before transcription, such as DNA methylation and chromatin accessibility, can help unravel causative regulatory variants by highlighting actively-transcribed regions of the genome, and the variants that sit within them. One of these factors is chromatin accessibility. Transcriptionally active genes, as well as active regulatory elements (such as enhancers), are found in regions of open chromatin (euchromatin); whereas inactive regions of the genome are typically much more densely compacted into a structure known as heterochromatin. Genome features found in euchromatin are therefore more accessible to transcription factors and other factors involved in gene expression, and so are more likely to influence traits of interest compared to factors located in inactive regions. Methods to assay chromatin accessibility include ChIP-seq [103], DNase-seq [104], and ATAC-seq [105].

Other factors acting during or after transcription provide intermediate phenotypes that can aid in understanding the underlying biological control of these traits [106]. One such factor is RNA-editing, i.e. direct enzymatic conversion of bases within the mRNA transcripts, with the most common form of editing in vertebrates being the conversion of adenosine nucleotides into inosine [107]. Biologically, RNA editing is involved in protection against dsRNA viruses [108] and in adaptation to different environmental conditions [109], and therefore has potential relevance to variation in animal health and in providing for animal adaptability to changing environments. RNA-editing QTL (edQTL) were initially identified in *Drosophila* [110], followed soon after by mice [111] and humans [112]. Recently, edQTL were reported for the first time within the bovine mammary gland [113], and subsequently used to characterise candidate causative genes underlying a milk yield QTL at the *CSF2RB/NCF4* locus [114]. That study highlighted the manner in which intermediate molecular phenotypes can be used to investigate mechanisms underlying FT-MIR predicted trait QTL, and exemplifies how other similarly novel molecular phenotypes can be applied.

#### Metabolomics

Absorbance levels at individual FT-MIR wavenumbers provide insights into the presence of particular chemical bonds in the sample and accordingly provide information as to the chemical composition of a milk sample. Analysing the chemical composition of a sample in more detail, using methodologies such as nuclear magnetic resonance (NMR) spectroscopy or mass spectroscopy (MS), yields the metabolome, i.e., a more complete set of all small molecules present in a tissue sample. Metabolomics can provide detailed information about enzymatic activity in the pathways that exist between gene expression and FT-MIR predicted traits, providing a near-terminal link in the chain of causality. For example, rumen volatile fatty acid (VFA) levels can provide information on measuring and controlling methane production [115]. Levels of VFAs in the rumen could therefore provide a proxy measurement for methane production. Identifying QTL that underlie variation in the concentrations of these metabolites could complement genetic signals identified using FT-MIR wavenumbers and FT-MIR based methane trait predictions, and facilitate selection of low-methane emitting animals.

## Conclusions

Over the last 100 years, milk composition phenotyping for dairy cattle has evolved from manual on-farm methods for determining yield and fat levels in milk, to high-tech analysis at centralised laboratories, with many novel FT-MIR predicted traits now being considered for incorporation into improvement programs. Multiple studies have demonstrated that the accuracy of FT-MIR predictions are strongly influenced by how well the variation in the prediction population is represented in the calibration population. Trait prediction accuracy is also strongly affected by how well instrument-specific measurement differences are accounted for, particularly when transferring calibration equations developed on one instrument to spectra collected on other instruments. Utilising FT-MIR data to generate proxies for novel traits has grown in popularity, however, compared to FT-MIR predictions of major milk components, there are relatively few studies of the genetics of other FT-MIR predicted traits, and even fewer of the genetics of the individual wavenumbers. This is despite the individual wavenumbers exhibiting additional genetic signal that is often not observed in FT-MIR predictions of major milk composition traits. Integrating results from GWAS applied to FT-MIR predicted traits and GWAS applied to individual wavenumbers with other molecular datasets could improve our understanding of the underlying biological systems controlling traits of interest. However, integration of these data sources also brings computational challenges due to the size and complexity of the datasets involved. Resolving the challenges of effectively integrating FT-MIR datasets with other omics data sources will require a mix of both bioinformatics and molecular biology approaches. Successfully consolidating these approaches promises to improve our knowledge of milk composition and enable the future enhancement of animal breeding programmes.

## Data Availability

Not applicable.

## Abbreviations

a_30_: Curd firmness after 30 min
a_60_: Curd firmness after 60 min
BHB: Beta-hydroxybutyrate
CH_4_: Methane
CMS: Casein micelle size
CY: Curd yield
EBV: Estimated breeding value
edQTL: RNA-editing QTL
eQTL: Expression quantitative trait loci
FT-MIR: Fourier-transform mid-infrared
GC: Gas chromatography
GWAS: Genome wide association study
HCT: Heat coagulation time
k_20_: Curd-firming time
LCFA: Long-chain fatty acids
MCFA: Medium-chain fatty acids
MD: Mahalanobis distance
MS: Mass spectroscopy
MUN: Milk urea nitrogen
NMR: Nuclear magnetic resonance
PLS: Partial least squares
PUFA: Polyunsaturated fatty acids
RCT: Rennet coagulation time
REC: Nutrient recovery
SCFA: Short-chain fatty acids
SFA: Saturated fatty acids
UFA: Unsaturated fatty acids
VFA: Volatile fatty acid
